# Supplementary material for: Timing of the Pubertal Growth Spurt and Prostate Cancer
Source: Cancers (Basel). 2021 Dec 12;13(24):6238. doi: 10.3390/cancers13246238 (PMC8699412; doi:10.3390/cancers13246238)

Supplementary – Timing of the pubertal growth spurt and prostate cancer

**Table of contents**	**Pages**
Material and methods	2
Results	3
Tables	4–7
Figures	8–9

Supplementary Material and methods

Covariates

BMI at age 8 and 20 years of age were estimated using all paired height and weight measurements in the age period of 6.5–9.5 years for BMI at 8, and in the age period of 17.5-22.0 years for BMI at 20, and were centered around 8 and 20 years of age, respectively, using a linear regression[14]. Height at 8 years of age was estimated using all height measurements between 6,5 and 9,5 years of age and age-adjusted with a linear regression model to age 8 years. For final height, the mean of available heights between 21 and 50 years of age in the Passport Register was used. We obtained information on education from linkage with demographic registers at Statistics Sweden and categorized the study subjects´ highest attained education into three levels (elementary school, secondary school or university level).

Supplementary Results

Adjusted analyses and competing risk

We also adjusted the main analyses with follow up starting at 20 years of age for available covariates (BMI at 8 and 20 years, height at 8 years and final height) with unaltered results (Table 4). Moreover, our findings were robust to adjustments for birthweight and level of education (Table 4). There were no interactions between age at PHV and any of the covariates ($p>0.05$ for all interaction terms).

To exclude the risk of bias related to different ethnicities, we re-performed the main analyses in the sub-cohort of men born in Sweden and with both parents born in Sweden. These analyses revealed mainly unaltered associations for Q5 vs Q2-4 of age at PHV and prostate cancer (sub-cohort born in Sweden: HR 0.84, 95% CI 0.73-0.96; entire cohort: HR 0.83, 95% CI 0.73-0.94).

Lastly, evaluation using cumulative incidence plots of prostate cancer and non-prostate cancer mortality did not indicate that there was competing non-prostate cancer mortality disturbing the present finding of decreased risk of prostate cancer for subjects with a late age at PHV compared with subjects with an age at PHV in the middle quintiles (Suppl Figure 2).

Supplementary Tables

Variable	Entire cohort (N=31,971)	Subjects without diagnosed prostate cancer (N=30,212)	Subjects diagnosed with high-risk[a] prostate cancer (N=449)	Subjects diagnosed with low-risk[b] prostate cancer (N=1,310)
Age at peak height velocity, years (SD)	14.1 (1.1)	14.1 (1.1)	14.0 (1.1)	14.0 (1.1)
BMI at 8 years, kg/m^2, mean (SD)	15.7 (1.4)	15.7 (1.4)	15.7 (1.3)	15.7 (1.3)
Height at 8 years, cm, mean (SD)	128.7 (5.2)	128.7 (5.2)	128.5 (5.2)	128.9 (5.2)
BMI at 20 years, kg/m^2, mean (SD)	21.4 (2.5)	21.4 (2.5)	21.3 (2.4)	21.1 (2.2)***
Final height, cm, mean (SD)	180.4 (6.4)	180.4 (6.4)	180.1 (6.3)	180.8 (6.4)*
Birthweight[c], kg, mean (SD)	3.58 (0.55)	3.58 (0.55)	3.58 (0.53)	3.61 (0.56)*
Country of birth[d], n(%)				
Sweden	26,749 (83.7)	25,230 (83.5)	372 (82.9)	1,147 (87.6)***
Other	5,222 (16.3)	4,982 (16.5)	77 (17.1)	163 (12.4)***
Level of education[e], n(%)				
Elementary school	5,263 (16.9)	5,000 (17.1)	65 (14.5)	198 (15.1)**
Secondary school	13,552 (43.6)	12,812 (43.7)	204 (45.4)	536 (40.9)**
University level	12,263 (39.5)	11,510 (39.3)	177 (39.4)	576 (44.0)**

Supplementary Table S1A Cohort demographics. Demographic data of the entire cohort, and for the subgroups of those diagnosed with prostate cancer and those not diagnosed with prostate cancer. Childhood refers to 8 years of age.
[a]High-risk or metastatic prostate cancer
[b]Low or intermediate-risk prostate cancer
[c]Birthweight was available in a sub-cohort, N=30,365.
[d]Country of birth categorized as Sweden when both study subject and both parents were born in Sweden, otherwise categorized as Other.
[e]Level of education available in a sub-cohort, N=31,078.
** $p < 0.01$ compared to subjects without diagnosed prostate cancer
*** $p < 0.001$ compared to subjects without diagnosed prostate cancer

Variable	Entire cohort (N=31,971)	Subjects with early pubertal timing, Q1 of age at PHV (N=6,394)	Subjects with average pubertal timing, Q2–4 of age at PHV (N=19,183)	Subjects with late pubertal timing, Q5 of age at PHV (N=6,394)
Age at peak height velocity, years (range)	14.1 (9.3–17.9)	12.5 (9.3–13.2)***	14.1 (13.2–15.0)	15.6 (15.0–17.9)***
BMI at 8 years, kg/m^2, mean (SD)	15.7 (1.4)	16.1 (1.5)***	15.7 (1.4)	15.5 (1.3)***
Height at 8 years, cm, mean (SD)	128.7 (5.2)	130.1 (5.2)***	128.8 (5.1)	127.2 (5.2)***
BMI at 20 years, kg/m^2, mean (SD)	21.4 (2.5)	22.2 (2.7)***	21.4 (2.4)	20.6 (2.4)***
Final height, cm, mean (SD)	180.4 (6.4)	179.6 (6.2)***	180.5 (6.3)	180.9 (6.6)***
Birthweight[a], kg, mean (SD)	3.58 (0.55)	3.56 (0.55)**	3.58 (0.55)	3.59 (0.55)
Country of birth[b], n(%)				
Sweden	26,749 (83.7)	5,267 (82.4)*	16,082 (83.8)	5,400 (84.5)
Other	5,222 (16.3)	1,127 (17.6)*	3,101 (16.2)	994 (15.5)
Level of education[c], n(%)				
Elementary school	5,263 (16.9)	1,066 (17.2)	3,136 (16.8)	1,061 (17.0)
Secondary school	13,552 (43.6)	2,647 (42.6)	8,114 (43.6)	2,791 (44.8)
University level	12,263 (39.5)	2,500 (40.2)	7,379 (39.6)	2,384 (38.2)

Supplementary Table S1B Cohort demographics. Demographic data of the entire cohort, and for the subgroups of quintiles (Q) of pubertal timing, measured as age at peak height velocity (PHV). Childhood refers to 8 years of age.
[a]Birthweight was available in a sub-cohort, N=30,365.
[b]Country of birth categorized as Sweden when both study subject and both parents were born in Sweden, otherwise categorized as Other.
[c]Level of education available in a sub-cohort, N=31,078.
* $p < 0.05$ compared to subjects with average pubertal timing (Q2–Q4)
** $p < 0.01$ compared to subjects with average pubertal timing (Q2–Q4)
*** $p < 0.001$ compared to subjects with average pubertal timing (Q2–Q4)

Base model further adjusted for:	HR (95% CI) for age at PHV, 5th quintile vs quintile 2-4		
	Prostate cancer	High-risk or metastatic prostate cancer	Low or intermediate risk prostate cancer
BMI at 8 years	0.82 (0.72–0.93)	0.73 (0.56–0.94)	0.86 (0.74–0.99)
BMI at 20 years	0.81 (0.71–0.92)	0.74 (0.57–0.95)	0.83 (0.72–0.97)
Height at 8 years	0.83 (0.73–0.94)	0.72 (0.55–0.93)	0.87 (0.75–1.00)
Final height	0.82 (0.73–0.93)	0.73 (0.56–0.94)	0.86 (0.74–0.99)
Birthweight†	0.82 (0.72–0.94)	0.72 (0.55–0.74)	0.86 (0.74–1.00)*
Level of education††	0.83 (0.73–0.94)	0.74 (0.57–0.94)	0.86 (0.74–1.00)*

Supplementary Table S2 Age at Peak Height Velocity and risk of prostate cancer adjusted for related covariates. Cox proportional hazards regression of the association between pubertal timing and prostate cancer, also stratified in high-risk or metastatic prostate cancer, and low or intermediate prostate cancer. Base model adjusted for birth year and country of birth, and further adjusted for one of the additional covariates at a time. Total N=31,971, 1,759 cases of prostate cancer, 449 cases of high-risk or metastatic prostate cancer, and 1,310 cases of low or intermediate risk prostate cancer at diagnosis. Each quintile represents 6,394 individuals except for Q3 that represents 6,395 individuals. High-risk prostate cancer defined as any of PSA >20 ng/mL, Gleason score 4+3=7 or above, T 3-4, N1 or M1.

†Birthweight available for a sub-cohort, N=30,365

††Level of educational available for a sub-cohort, N=31,078

*$p < 0.05$

	HR (95% CI) for age at PHV	
Quintiles	High-risk prostate cancer	Low or intermediate risk prostate cancer
Q1	1.20 (0.91–1.57)	1.03 (0.90–1.18)
Q2-4	Ref	Ref
Q5	0.69 (0.52–0.93)	0.86 (0.75–0.99)

Supplementary Table S3 Age at Peak Height Velocity and risk of high- or low-risk prostate cancer categorized by Gleason score.
Cox proportional hazards regression of the association between pubertal timing and prostate cancer stratified in high-risk (n=355) prostate cancer, and low or intermediate (n=1,404) prostate cancer at diagnosis. Adjusted for birth year and country of birth. Total N=31,971. Each quintile represents 6,394 individuals except for Q3 that represents 6,395 individuals. High-risk or metastatic prostate cancer defined as Gleason score 4+3=7 or higher, low or intermediate risk defined as Gleason 3+4=7 or lower.

Supplementary Figures

Supplementary Figure S1 Distribution of prostate cancer and high-risk or metastatic prostate cancer across quintiles of age at peak height velocity

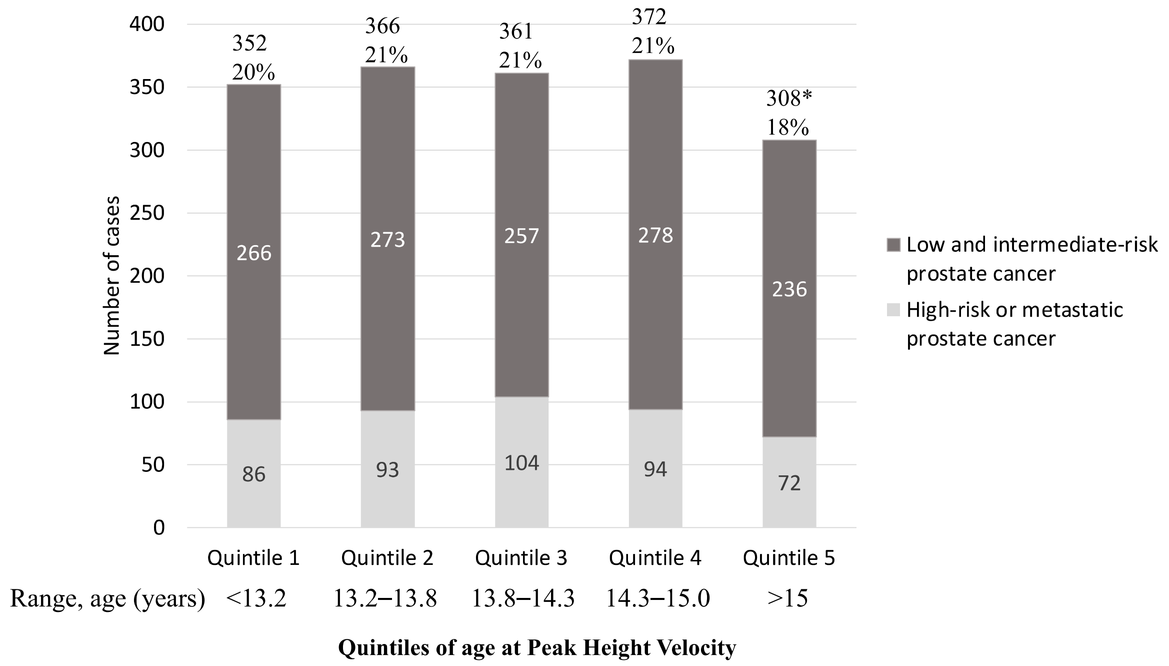

Individuals were categorized into quintiles according to age at peak height velocity (PHV). Total n=31,971; quintiles 1, 2, 4 and 5 (Q1, Q2, Q4 and Q5) n=6,394 per quintile, quintile 3 (Q3) n=6,395. Total number of cases n=1,759, high risk or metastatic cancer n=449 (defined as any of PSA >20, Gleason score 4+3=7 or higher, T 3-4, N1 or M1). Number (% of total) of prostate cancer cases for each quintile is indicated on top of each bar. Light grey areas represent high risk or metastatic cancer and dark grey areas represent low or intermediate risk cases (not fulfilling high risk or metastatic prostate cancer criteria).

* P<0.05 Q5 versus Q2-4 (chi square test).

Supplementary Figure S2 Cumulative Incidence Plots of prostate cancer events (A), and non-prostate cancer mortality (B)

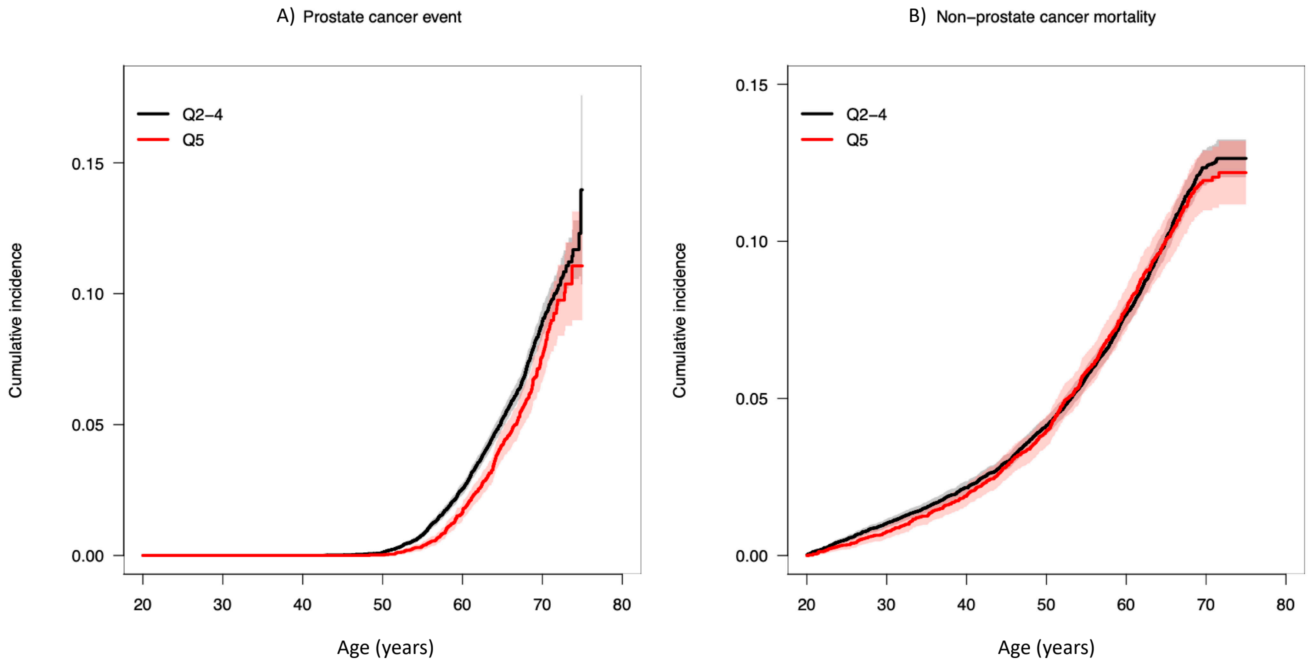

Supplement: Supplementary file 1 [file cancers-13-06238-s001.zip › cancers-1458056-supplementary.pdf]
